# The Relation of Accelerometer-Measured Physical Activity and Serum Uric Acid Using the National Health and Nutrition Survey (NHANES) 2003–2004

**DOI:** 10.3389/fspor.2021.775398

**Published:** 2022-01-12

**Authors:** Isaac D. Smith, Leanna M. Ross, Josi R. Gabaldon, Nicholas Holdgate, Carl F. Pieper, Tony C. Ning, William E. Kraus, Kim M. Huffman

**Affiliations:** ^1^Department of Medicine, Duke University School of Medicine, Duke University Hospital, Durham, NC, United States; ^2^Division of Rheumatology and Immunology, Duke University School of Medicine, Duke University Hospital, Durham, NC, United States; ^3^Duke Molecular Physiology Institute, Duke University, Durham, NC, United States; ^4^Oncology and Hematology Business Unit, Syneos Health Global Headquarters, Morrisville, NC, United States; ^5^Lowcountry Rheumatology, Charleston, SC, United States; ^6^Department of Biostatistics and Bioinformatics, Duke University School of Medicine, Durham, NC, United States; ^7^Triangle Orthopedic Associates, Durham, NC, United States; ^8^Division of Cardiology, Duke University School of Medicine, Durham, NC, United States

**Keywords:** exercise, accelerometer, uric acid, gout, hyperuricemia

## Abstract

**Objective:** Gout is a crystal-induced inflammatory arthritis caused by elevated uric acid. Physical activity has the potential to reduce serum uric acid (SUA), thus improving the disease burden of gout. In this study, we examined the association of objectively-measured physical activity and SUA.

**Methods:** A cross-sectional study was conducted using survey, laboratory, and accelerometer data from the 2003–2004 National Health and Nutrition Examination Survey (NHANES). SUA concentrations (mg/dL) were obtained during an initial exam, and then physical activity (kCal/day) was measured with 7 days of ActiGraph accelerometry in participants (*n* = 3,475) representative of the ambulatory, non-institutionalized US civilian population. Regression, including restricted cubic splines, was used to assess the relation of physical activity and SUA in bivariate and adjusted models. Covariates included age, gender, race/ethnicity, alcohol use, body mass index, renal function, and urate-lowering therapy.

**Results:** In the bivariate model, physical activity was correlated with SUA concentrations and included a non-linear component (*p* < 0.01). In the adjusted model, linear splines were employed with a node at the SUA nadir of 5.37mg/dL; this occurred at 703 kCal/day of physical activity. The association of physical activity and SUA was negative from 0 to 703 kCal/day (*p* = 0.07) and positive >703 kCal/day (*p* < 0.01 for the change in slope).

**Conclusion:** Physical activity and SUA are associated in a non-linear fashion, with a minimum estimated SUA at 703 kCal/day of objectively-measured physical activity. These findings raise intriguing questions about the use of physical activity as a potential adjunctive therapy in patients with gout, and further interventional studies are needed to elucidate the effects of moderate intensity exercise on SUA concentrations.

## Introduction

Gout is a crystal-induced inflammatory arthritis caused by excess uric acid. Both gout and elevated serum uric acid (SUA) concentrations are associated with metabolic syndrome, a disorder characterized by central obesity, dyslipidemia, hypertension, and glucose intolerance (Tsouli et al., 2006). In the United States (US), the prevalence of metabolic syndrome is significantly higher in patients with gout (63%) than in the general population (25%) (Choi et al., 2007). Moreover, both elevated SUA concentrations and metabolic syndrome are associated with hyperinsulinemia, which may lead to decreased renal excretion of uric acid (Quiñones Galvan et al., 1995; Perez-Ruiz et al., 2015; Toyoki et al., 2017).

Even at moderate intensity, physical activity improves insulin sensitivity and decreases concentrations of fasting insulin (Houmard et al., 2004), which may increase renal uric acid excretion (Quiñones Galvan et al., 1995; Perez-Ruiz et al., 2015; Toyoki et al., 2017). Regular physical activity may also help mitigate the increased risk of cardiovascular disease (CVD) and mortality in patients with gout (Chen et al., 2015; Disveld et al., 2018; Fiuza-Luces et al., 2018), though little is known about the ideal level of exercise in this patient population. Previous studies have demonstrated professional athletes, as well individuals with higher levels of fitness, have lower SUA concentrations than healthy controls (Church et al., 2002; Lippi et al., 2004). Self-reported physical activity has also been associated with lower odds of hyperuricemia among healthy individuals (Park et al., 2019), and in one interventional study of obese Japanese men, moderate physical activity was associated with lower uric acid concentrations (Nishida et al., 2011).

Currently, there are no American College of Rheumatology guidelines regarding physical activity levels in patients with gout (Fitzgerald et al., 2020), and to our knowledge, no studies have quantitatively evaluated the relation of objectively measured physical activity and SUA concentrations in the US population. We hypothesize that greater amounts of physical activity reduce SUA concentrations, and this is of high clinical relevance for patients with gout and hyperuricemia. Here, our objective was to evaluate whether objectively measured physical activity is correlated with lower SUA concentrations in a large, nationally representative, cross-sectional population sample.

## Materials and Methods

### Study Population

A cross-sectional study was conducted using data from the 2003–2004 National Health and Nutrition Examination Survey (NHANES). NHANES refers to a series of cross-sectional studies examining the health and nutritional status of adults and children in the US. The 2003–2004 NHANES study population (*N* = 12,761) was selected using a stratified, multistage probability sampling design and included a representative sample of the noninstitutionalized US civilian population with certain oversampled populations (adolescents, patients ≥60 years old, African-Americans, and Mexican-Americans) (Centers for Disease Control Prevention, 2005). Our study included ambulatory participants ≥20 years-old with reported SUA concentrations who also participated in the accelerometry portion of NHANES 2003–2004 (*n* = 3,475).

### Assessment of Covariates

Demographic data including age, sex, and race/ethnicity were self-reported by participants and were assessed at the time the survey was administered. Gout status was not assessed as part of the NHANES 2003–2004 survey, but the use of urate-lowering medications (i.e., allopurinol and probenecid) during the previous 1 month was obtained by research personnel during the household interview. Allopurinol use and probenecid use were included in our adjusted analysis as categorical variables (yes/no). Per NHANES survey criteria, alcohol use was also defined categorically (yes/no) based on whether survey participants reported ≥12 drinks in the past 1 year. Body mass index (BMI) was calculated using the height and weight obtained by physical exam and was evaluated as a continuous variable. Blood samples were collected at mobile examination centers, and our study included SUA concentrations and renal function, assessed by serum creatinine (mg/dL). These values were evaluated as continuous variables and were measured using a Beckman Synchron LX20 chemistry analyzer as part of a standard biochemistry profile.

### Physical Activity Assessment

Following the initial evaluation described above, physical activity was assessed with seven days of accelerometry. Participants were instructed to wear an Actigraph accelerometer (model 7164) during all waking hours of the day, except while bathing or swimming. The device was attached to their right hip by an elastic belt. The device recorded vertical acceleration as “counts,” which correspond to intensity of physical activity. The device was checked using an ActiGraph calibrator, and data were recorded in 1-min epochs. Non-wear time was defined as 20 or more consecutive minutes of zero counts; non-wear time was excluded from the analysis. To prevent aberrantly high activity measures, days where recorded activity was >40,000 kilocalories (kCal) were also excluded. A day was considered valid when data were available for at least 12 h in a 24-h period, and the final dataset included only those participants with at least four valid days (>12 h/24-h period) of wear time.

Accelerometer counts were converted to energy expenditure (kCal/min) using algorithms provided by the National Cancer Institute (http://appliedresearch.cancer.gov/nhanes_pam/) as well as the CALERIE accelerometer study (Sloane et al., 2009). Specifically, for count-to-energy expenditure conversions, the work-energy equation was used at lower activity intensity (counts per 1-min epoch <1,952) and the Freedson energy equation was used at moderate and high intensities (counts per 1-min epoch >1,952). Average daily energy expenditure (kCal/day) was determined for all individuals.

### Statistical Analysis

Adjusting for the complex sampling design employed by the NHANES 2003–2004 survey, multivariable regression was used to evaluate the relation of objectively-measured physical activity using accelerometer data and SUA concentrations. We initially assessed a bivariate model to directly compare the relation of physical activity and SUA concentrations. This model was subsequently adjusted for age, sex, race/ethnicity, alcohol use, BMI, serum creatinine, and the use of urate-lowering medications. The relationship between physical activity and SUA was assumed to have both linear and non-linear components. Restricted cubic splines (Harrell, 2015) were initially employed to investigate the functional form of the non-linear component, with follow-up inspection of the resulting plots to assess if a simpler parameterization (e.g., higher order polynomials, linear splines) would be revealed. The final model would be composed of all covariates, and both a linear and a non-linear component–the later suggested by the prior step. Secondary analyses were also performed to assess possible interactions between physical activity and sex, and physical activity and BMI (≤ 25 or >25 kg/m^2^). All *p*-values were two-sided, and *p* < 0.05 was considered to be significant for all tests. All statistical analyses were conducted using SAS software (V.9.3, SAS Institute, Cary, NC, USA).

## Results

The characteristics of the study population are summarized in Table 1. The median [interquartile range (IQR)] SUA concentration among participants was 5.22 (4.31–6.21) mg/dL, and 0.65% of participants reported using urate-lowering medications during the previous month. The median (IQR) energy expenditure from physical activity was 403 (262–604) kCal/day.

**Table 1:** Subject characteristics.

**Characteristic**	***N* = 3,475**
Age, median (IQR)	45.3 (33.0–57.8)
Sex, female %	50.9%
Race/ethnicity %	
White	73.5%
Black	9.8%
Mexican American	7.7%
Other Hispanic	3.6%
Other	5.4%
BMI, kg/m^2^, median (IQR)	27.3 (24.0–31.4)
Physical activity (kCal/day)	
Mean ± SD	468 ± 291
Median (IQR)	403 (262–604)
Creatinine, mg/dL, median (IQR)	0.81 (0.69–0.96)
Serum uric acid, mg/dL, median (IQR)	5.22 (4.31–6.21)
Uric acid ≤ 4 mg/dL	17.3%
Uric acid 4.1–6mg/dL	52.5%
Uric acid 6.1–8mg/dL	27.1%
Uric acid >8mg/dL	3.2%
Medication use %	0.65%
Allopurinol^†^	0.61%
Probenecid	0.04%
Alcohol use^‡^ %	72.3%

*BMI, body mass index; SD, standard deviation; IQR, interquartile range*.

† *Febuxostat was not approved in the US until 2009*.

‡ *Alcohol use defined per survey results as >12 drinks per 1 year*.

**Figure 1:**
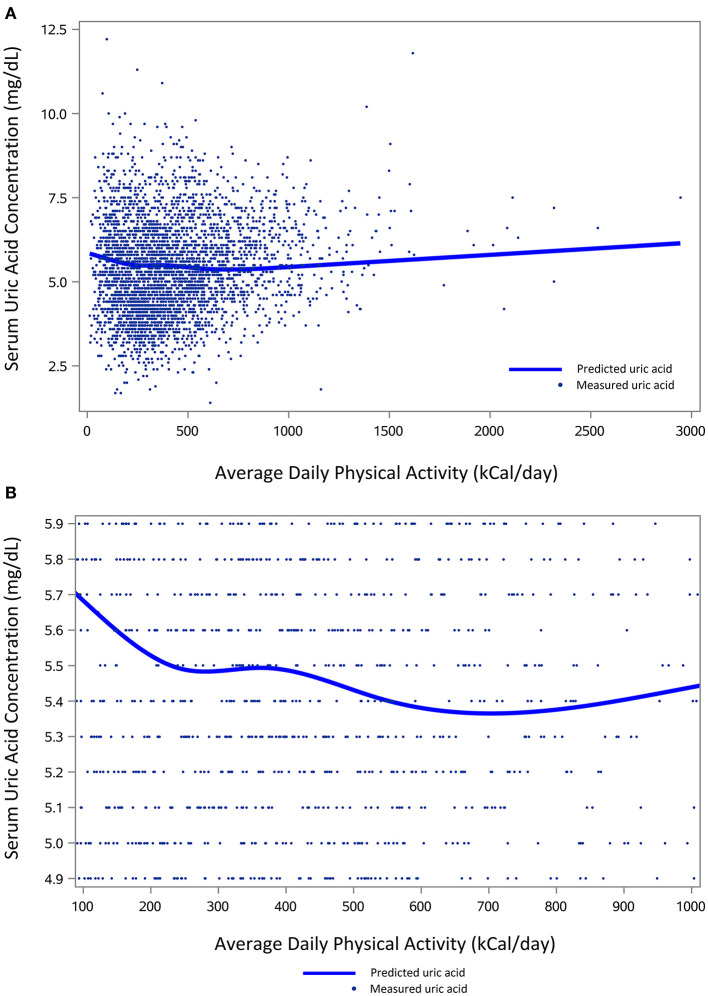
The relation of physical activity and serum uric acid concentrations. **(A)** Depicts measured and predicted uric acid concentrations (mg/dL) across the entire range of average daily physical activity (kCal/day). **(B)** Shows a more focused view of predicted uric acid concentrations for the middle 95% of the population (median ± 47.5%) based on average daily physical activity. Predicted uric acid levels for both figures are based on the same multivariable analysis, adjusted for age, gender, race/ethnicity, alcohol use, Body Mass Index, serum creatinine, and use of urate-lowering medications.

Physical activity, reported as energy expenditure in kCal/day, was significantly associated with SUA concentrations in our initial bivariate model (*p* < 0.01), and follow-up analyses with restricted cubic splines identified a non-linear component to the relationship. After controlling for age, sex, race/ethnicity, alcohol use, BMI, renal function, and the use of urate-lowering therapy, the graphical appearance of the adjusted model suggested a V-shaped relationship between physical activity and SUA concentration (Figures 1A,B), with an asymptote at ~700 kCal/day. Thus, the relationship was modeled using linear splines.

When the linear and spline terms were included in the controlled model, the results indicated a negative correlation (β = −0.00046, *p* = 0.07) between units of physical activity (i.e., 1 kCal/day) and SUA from 0 to 703 kCal/day and a positive correlation (β = 0.00038, *p* < 0.01) above 703 kCal/day. Using mean values for our covariates, predicted SUA concentrations were as high as 5.84 mg/dL among individuals with minimal activity levels, and the SUA nadir of 5.37 mg/dL was noted to occur at 703 kCal/day of physical activity. The difference between the corresponding slope from 0 to 703 kCal/day and the slope >703 kCal/day was statistically significant (*p* < 0.01).

In addition to physical activity levels, age, sex, race/ethnicity, BMI, renal function, and use of allopurinol (but not probenecid) were all significantly related to SUA concentrations (*p* < 0.05 for all covariates). There was no significant interaction between the physical activity term and sex or BMI (≤ 25 or >25 kg/m^2^) in relation to SUA concentrations in our secondary analyses.

## Discussion

In this cross-sectional study of a large, nationally representative sample of US adults, the relation of physical activity and SUA concentrations were associated in a non-linear fashion, with a minimum estimated SUA at 703 kCal/day of objectively-measured physical activity in our adjusted model. Physical activity and SUA concentrations were negatively correlated from 0 to 703 kCal/day and positively correlated >703 kCal/day. In participants with minimal activity levels, predicted SUA concentrations based on mean covariate values were as high as 5.84 mg/dL, and the SUA nadir of 5.37 mg/dL was observed at 703 kCal/day of physical activity. To our knowledge, no studies have quantitatively evaluated the relation of objectively measured physical activity and SUA concentrations in the US population, and overall, the results from this study appear to suggest a beneficial association between physical activity and SUA.

Our results support findings from prior investigations in unique populations, or using self-reported physical activity. In obese male factory workers in Japan, moderate, but not light or vigorous, intensity physical activity was associated with lower SUA concentrations (Nishida et al., 2011). Similar results were noted in a study of South Korean men and women, where self-reported participation in regular health-enhancing physical activity was associated with lower odds of hyperuricemia (Park et al., 2019).

Though we cannot determine causality in our cross-sectional study, we hypothesize that the reduced SUA concentrations observed with physical activity may be related to changes in insulin sensitivity. Physical activity reduces insulin resistance and decreases fasting insulin levels, particularly in individuals who engage in regular moderate aerobic exercise (Houmard et al., 2004). Favorable insulin changes related to physical activity may increase renal uric acid excretion (Quiñones Galvan et al., 1995; Perez-Ruiz et al., 2015; Toyoki et al., 2017), resulting in reduced SUA concentrations, and these observations may be highly relevant for patients with hyperuricemia. While further prospective studies are needed to confirm our findings in patients with gout, physical activity not only has the potential to reduce SUA concentrations but may also help prevent cardiovascular disease in this high-risk population (Camhi et al., 2011; Fiuza-Luces et al., 2018).

Through our previous work in patients with pre-diabetes, we have found that moderate intensity aerobic exercise training produces superior improvements in insulin sensitivity compared to vigorous intensity activities (Slentz et al., 2016), and this may be one potential reason why an attenuated SUA effect was observed for participants averaging >703 kCal/day. Other possible explanations for the attenuated effect of higher levels of physical activity on SUA concentrations include dietary considerations (higher purine intake or fructose-sweetened beverage consumption) (Hak and Choi, 2008), increased uric acid release from muscle damage and protein catabolism (Hammouda et al., 2012; Jamurtas et al., 2018), and competitive inhibition of uric acid excretion due to increased lactic acid production (1967;Neumayr et al., 2003).

The strengths of our study are the large, nationally representative population provided by the NHANES dataset and the use of accelerometry data to objectively measure physical activity. We also recognize a number of limitations primarily related to the cross-sectional nature of this study. Although participants wore accelerometers for 1 week, in the absence of an intervention, this provides only a cross-sectional assessment of regular physical activity. Thus, we were unable to determine causality and the mechanism(s) of the observed associations. Also, only one SUA measurement was performed, and this occurred prior to the objective physical activity assessment period. Because SUA exhibits biological variability, additional assessments of SUA would have improved SUA accuracy. A gout diagnosis was not included in the list of medical conditions captured in the 2003–2004 NHANES questionnaire, and all patient sociodemographic and medication information was self-reported.

## Conclusion

In this cross-sectional study of the US population, physical activity and SUA concentrations were associated in a non-linear fashion, with a minimum estimated SUA at 703 kCal/day of objectively-measured physical activity. These findings raise intriguing questions about the use of physical activity as a potential adjunctive therapy in patients with gout, and further interventional studies are needed to elucidate the effects of moderate intensity exercise on SUA concentrations.

## Data Availability Statement

The datasets analyzed for this study can be found in the 2003–2004 National Health and Nutrition Examination Survey (NHANES) at https://wwwn.cdc.gov/nchs/nhanes/continuousnhanes/default.aspx?BeginYear=2003. Further inquiries can be directed to the corresponding author.

## Ethics Statement

The NHANES 1999–2004 study involving human participants was reviewed and approved by the NCHS Research Ethics Review Board (protocol #98-12 for NHANES 1999–2004). The participants provided their written informed consent to participate in the NHANES study. As the present analysis only uses de-identified data from the NHANES 2003–2004 dataset, the current study was designated as exempt human-subjects research and was approved by the Institutional Review Board of Duke University Hospital (IRB Pro00030650).

## Author Contributions

IS, LR, JG, NH, CP, TN, WK, and KH contributed to the conception and design of the study. CP, NH, and TN organized the database. CP performed the statistical analysis, produced the tables, and figures for the manuscript. IS, LR, and JG wrote the first draft of the manuscript. NH, TN, WK, and KH edited multiple drafts of the manuscript. All authors contributed to manuscript revision, read, and approved the submitted version.

## Funding

This project was funded through NIH/NIA grant #P30AG028716.

## Conflict of Interest

The authors declare that the research was conducted in the absence of any commercial or financial relationships that could be construed as a potential conflict of interest.

## Publisher's Note

All claims expressed in this article are solely those of the authors and do not necessarily represent those of their affiliated organizations, or those of the publisher, the editors and the reviewers. Any product that may be evaluated in this article, or claim that may be made by its manufacturer, is not guaranteed or endorsed by the publisher.

## References

[B1] (1967). Lactic acid and hyperuricemia of faulty renal urate transport. JAMA. 199, 121–122. 10.1001/jama.199.2.121b 5225152

[B2] Camhi S. M. Sisson S. B. Johnson W. D. Katzmarzyk P. T. Tudor-Locke C. (2011). Accelerometer-determined moderate intensity lifestyle activity and cardiometabolic health. Prev. Med. 52, 358–60. 10.1016/j.ypmed.2011.01.030 21300082

[B3] Centers for Disease Control Prevention. (2005). NHANES 2003-2004 Pubic Data General Release File Documentation. Available online at: https://www.cdc.gov/NCHS/data/nhanes/nhanes_03_04/general_data_release_doc_03-04.pdf (accessed August 1, 2021).

[B4] Chen J. H. Wen C. P. Wu S. B. Lan J. L. Tsai M. K. Lee J. H. (2015). Attenuating the mortality risk of high serum uric acid: the role of physical activity underused. Ann. Rheum. Dis. 74, 2034–42. 10.1136/annrheumdis-2014-205312 25053714

[B5] Choi H. K. Ford E. S. Li C. Curhan G. (2007). Prevalence of the metabolic syndrome in patients with gout: the Third National Health and Nutrition Examination Survey. Arthritis Rheum. 57, 109–15. 10.1002/art.22466 17266099

[B6] Church T. S. Finley C. E. Earnest C. P. Kampert J. B. Gibbons L. W. Blair S. N. (2002). Relative associations of fitness and fatness to fibrinogen, white blood cell count, uric acid and metabolic syndrome. Int. J. Obes. Relat. Metab. Disord. 26, 805–13. 10.1038/sj.ijo.0802001 12037651

[B7] Disveld I. J. Fransen J. Rongen G. A. Kienhorst L. B. Zoakman S. Jannsens H. J. Crystal-proven gout characteristic gout severity factors are associated with cardiovascular disease. J. Rheumatol. (2018) 45, 858–863. 10.3899/jrheum.170555 29657151

[B8] Fitzgerald J. D. Dalbeth N. Mikuls T. Brignardello-Petersen R. Guyatt G. Abeles A. M. (2020). American college of rheumatology guideline for the management of gout. Arthritis Care Res. (2020) 72, 744–760. 10.1002/acr.24375 32391934 PMC10563586

[B9] Fiuza-Luces C. Santos-Lozano A. Joyner M. Carrera-Bastos P. Picazo O. Zugaza J. L. (2018). Exercise benefits in cardiovascular disease: beyond attenuation of traditional risk factors. Nat. Rev. Cardiol. 15, 731–43. 10.1038/s41569-018-0065-1 30115967

[B10] Hak A. E. Choi H. K. (2008). Lifestyle and gout. Curr. Opin. Rheumatol. 20, 179–86. 10.1097/BOR.0b013e3282f524a2 18349748

[B11] Hammouda O. Chtourou H. Chaouachi A. Chahed H. Ferchichi S. Kallel C. (2012). Effects of short-term maximal exercise on biochemical markers of muscle damage, total antioxidant status, and homocysteine levels in football players. Asian J. Sports Med. 3, 239–46. 10.5812/asjsm.34544 23342222 PMC3525820

[B12] Harrell F. E. (2015). Regression modeling strategies with applications to linear models, logistic and ordinal regression, and survival analysis. 2nd ed. Cham, Switzerland: Springer International AG. 10.1007/978-3-319-19425-7

[B13] Houmard J. A. Tanner C. J. Slentz C. A. Duscha B. D. (2004). McCartney JS, Kraus WE. Effect of thevolume and intensity of exercise training on insulin sensitivity. J. Appl. Physiol. 96, 101–6. 10.1152/japplphysiol.00707.2003 12972442

[B14] Jamurtas A. Z. Fatouros I. G. Deli C. K. Georgakouli K. Poulios A. Draganidis D. (2018). The effects of acute low-volume HIIT and aerobic exercise on leukocyte count and redox status. J. Sports Sci. Med. 17, 501–8. 30116124 PMC6090390

[B15] Lippi G. Brocco G. Franchini M. Schena F. Guidi G. (2004). Comparison of serum creatinine, uricacid, albumin and glucose in male professional endurance athletes compared with healthy controls. Clin. Chem. Lab. Med. 42, 644–7. 10.1515/CCLM.2004.110 15259381

[B16] Neumayr G. Pfister R. Hoertnagl H. Mitterbauer G. Getzner W. Ulmer H.. (2003). The effect of marathon cycling on renal function. Int. J. Sports Med. 24, 131–137. 10.1055/s-2003-38205 12669260

[B17] Nishida Y. Iyadomi M. Higaki Y. Tanaka H. Hara M. Tanaka K. (2011). Influence of physical activity intensity and aerobic fitness on the anthropometric index and serum uric acid concentrations in people with obesity. Intern. Med. 50, 2121–8. 10.2169/internalmedicine.50.5506 21963729

[B18] Park D. Y. Kim Y. S. Jin Ryu S. H. Jin Y. S. (2019). The association between sedentary behavior, physical activity, and hyperuricemia. Vasc. Health Risk Manag. 15, 291–9. 10.2147/VHRM.S200278 31616149 PMC6698593

[B19] Perez-Ruiz F. Aniel-Quiroga M. A. Herrero-Beites A. M. Chinchilla S. P. Erauskin G. G. Merriman T. (2015). Renal clearance of uric acid is linked to insulin resistance and lower excretionof sodium in gout patients. Rheumatol. Int. 35, 1519–24. 10.1007/s00296-015-3242-0 25763991

[B20] Quiñones Galvan A. Natali A. Simona B. Fascerra S. Sanna G. Ciociaro D. (1995). Effect of insulin on uric acid excretion in humans. Am. J. Physiol. Endocrinol. Metabol. 268, E1–5. 10.1152/ajpendo.1995.268.1.E1 7840165

[B21] Slentz C. Bateman L. Willis L. Granville E. Piner L. Samsa G.. (2016). Effects of exercise training alone vs a combined exercise and nutritional lifestyle intervention on glucose homeostasis in prediabetic subjects: a randomised controlled trial. Diabetologia. 59, 2088–98. 10.1007/s00125-016-4051-z 27421729 PMC5026926

[B22] Sloane R. Snyder D. C. Demark-Wahnefried W. Lobach D. Kraus W. E. (2009). Comparing the 7-day physical activity recall with a triaxial accelerometer for measuring time in exercise. Med. Sci. Sports Exerc. 41, 1334–40. 10.1249/MSS.0b013e3181984fa8 19461530 PMC2686118

[B23] Toyoki D. Shibata S. Kuribayashi-Okuma E. Xu N. Ischizawa K. Hosoyamada M. (2017). Insulin stimualates uric acid reabsorption via regulating urate transporter 1 and ATP-binding cassette subfamily G member 2. Am. J. Physiol. Renal. Rhysiol. 313, F826–34. 10.1152/ajprenal.00012.2017 28679589

[B24] Tsouli S. G. Liberopoulos E. N. Mikhailidis D. P. Athyros V. G. Elisaf M. S. (2006). Elevated serum uric acid levels in metabolic syndrome: an active component or an innocent bystander? Metabolism. 55, 1293–1301. 10.1016/j.metabol.2006.05.013 16979398

